# Ang Ating Mata: Disparities in Eye Health Knowledge, Attitudes and Practices among Older Adult Filipino-Americans in the San Francisco Bay Area Counties

**DOI:** 10.1007/s10903-022-01371-3

**Published:** 2022-06-29

**Authors:** Marycon Chin Jiro, Michael Sigua, Susan L. Ivey, Marlon Maus, Lauren Hennein, Migel Dio, Jennifer Cocohoba

**Affiliations:** 1 Department of Medicine, University of California, San Francisco, CA USA; 2 School of Public Health, University of California, Berkeley, CA USA; 3 Department of Ophthalmology, Boston Children’s Hospital, Harvard Medical School, Boston, MA USA; 4 Department of Clinical Pharmacy, University of California, San Francisco, CA USA

**Keywords:** Filipino-American, Cultural messaging, Eye care, knowledge, attitudes, and practices, Eye diseases, Health Disparities

## Abstract

Filipino-Americans are the third largest Asian-American population, with a median age of 44. However, there is limited literature focusing on the group’s ophthalmic care engagement. Timely eye examinations and outreach are necessary to reduce visual impairment in this older community. To assess eye care knowledge, attitudes, and practices, we conducted a cross-sectional study surveying Filipino-Americans within the nine San Francisco Bay Area counties. Associations between primary outcomes and sociodemographic factors were analyzed using chi-squared analysis and student’s T-test. In our convenience sample of 256 surveys, a majority of participants are receiving appropriate eye care; those that lacked health and eye insurance, immigrated and are lower income did not receive optimal eye care. Study participants also demonstrated a lack of awareness of eye diseases and risk factors. Our results suggest that culturally sensitive eye health education materials are lacking and should be made accessible for this large and rapidly growing population.

**Supplementary Information:**

The online version contains supplementary material available at 10.1007/s10903-022-01371-3.

## Background

In the United States (U.S.), approximately 12 million people 40 years and older have vision impairment, including one million who are blind and eight million who have uncorrected refractive error [1, 2]. Visual impairment and blindness have an annual financial impact estimated at $12,000–24,000 per patient, which is almost two-fold the cost for non-blind patients [3, 4]. Poor eye health negatively affects quality of life as well as restricts equitable access to and achievement in society [5]. This widens disparities already disproportionately affecting low-income, older, and minority U.S. residents [6–8]. As the U.S. population continues to age, impaired vision will only become a bigger public health concern.

Filipino-Americans comprise 3.5 million of the U.S. population with a median age of 44 years old born in the U.S. and 51 years old among foreign-born Filipinos. This older, growing group is the largest Southeast Asian and third largest Asian-American population [9], with the greater San Francisco (SF) metropolitan area having the second largest Filipino-American population. While studies have highlighted the high prevalence of cardiometabolic diseases within the Filipino-American population, there is markedly limited literature assessing other conditions such as the state of ophthalmic health. It was found that other ethnic minority groups in the United States receive less eye health outreach and eye care [10–13], but it is unknown whether Filipino-Americans also receive less eye care and outreach as well. This is of concern because risk factors that disproportionately affect Filipino-Americans, such as high blood pressure, diabetes, and structural inequities [14, 15], place this population at increased risk of overall worsened eye health. Additionally, three studies conducted at community-based comprehensive, private ophthalmology clinics in Northern California demonstrated that the prevalence of all forms of diabetic retinopathy (DR) among Filipinos was approximately twice as high compared to the prevalence among Caucasians and that Filipino-American patients had significantly higher prevalence of narrow anterior chamber angles of the eye, a risk factor for glaucoma [16–18].

To gain a cross-sectional snap-shot of behavioral, knowledge and practice patterns, we conducted a cross-sectional Knowledge, Attitudes and Practices (KAP) survey of Filipino-Americans aged 40 and older within the nine SF Bay Area counties to assess eye care knowledge, attitudes, and health seeking practices. KAP surveys use health behavior change theory useful in measuring and revealing new information about a target population’s perceptions and revealing misconceptions or misunderstandings that may represent obstacles to future interventions and policy [19].

## Methods

### Participant Outreach

**Fig. 1:**
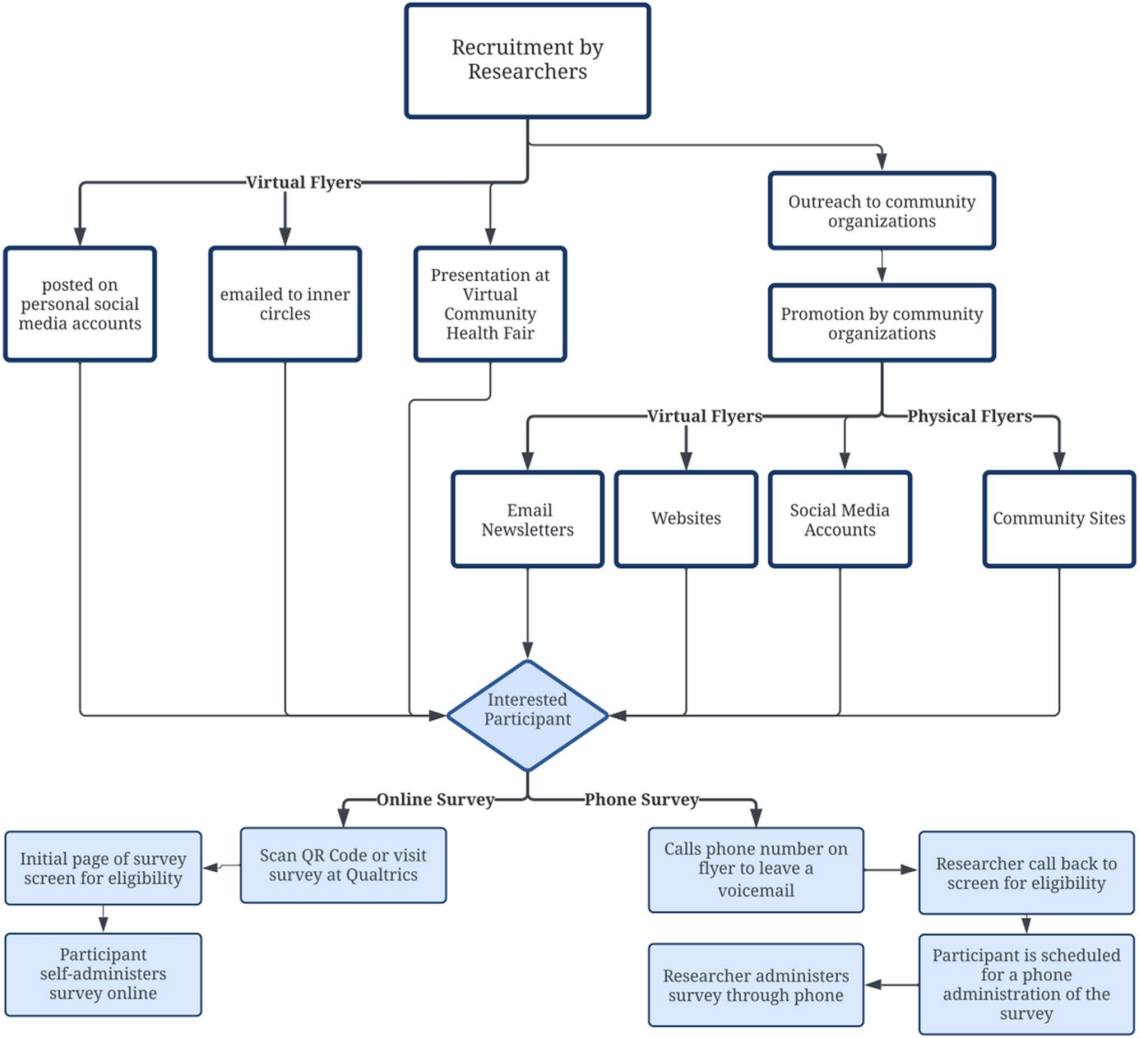
Participant recruitment process. Participants were recruited by researchers along with the help of community organizations who promoted the study to their client base through virtual and physical flyers

Our inclusion criteria were self-identified Filipino-Americans aged 40 or older fluent in English and/or Tagalog residing in the nine Bay Area counties (SF, San Mateo, Alameda, Contra Costa, Santa Clara, Marin, Napa, Sonoma and Solano). In compliance with shelter-in-place COVID-19 pandemic protocols and to capture different demographics, participants were recruited via telephone and online in partnership with SF Bay Area Filipino-American organizations (Fig. 1). Both modalities were made available in either English or Tagalog Filipino. Verbal or online consent was obtained. The first 250 participants of the survey were compensated with a $5 gift card. After survey completion, participants were provided a list of eye health resources created by the research team in English or Tagalog.

### Data Collection and Measures

The Filipino-American Eye Health Survey contained 54 questions and was made available between July 14, 2020 and August 12, 2020, using QualtricsXM (Online Appendix 1). The survey was developed by adapting questions from a 2005 KAP Survey [20]. A Short Acculturation Scale for Filipino-Americans (ASASFA), a validated 12-item questionnaire measuring level of acculturation among Filipino-Americans, was included. The tool measures three factors of acculturation using a 5-point Likert scale: language use, media language preference and ethnic social relation. Each item is scored according to the value assigned to the response. The lowest total score is 12 and the highest total score is 60. The possible mean scores for the total scale and subscales range from 1 to 5. The higher mean scores indicate a higher level of acculturation toward American culture and the lower mean scores indicate less acculturation. The ASASFA has been used in other studies focused on Filipino-American health issues [21–23].

A pilot test was performed to assess for validity and consistency in our study population. Individuals participating in the pilot test were asked to assess appropriateness, wording, and comprehension of the questionnaire. Data from the pretest were not included in the final analysis.

Since this was a convenience sample, we accepted all completed surveys, then disregarded those not in our inclusion criteria. Participants were screened for eligibility using a standard series of questions. For online surveys, those who did not meet all criteria were denied access to the rest of the survey. To further exclude suspicious automated responses, a captcha box on the online survey was generated. Lastly, we included only the first entry by the participant for responses that used the same email twice. Surveys recorded gender, age, demographics, self-reported diagnoses, eye health attitudes, knowledge, and eye care practices.

### Data Analysis

All data were de-identified during the data analysis stage. Participant demographics, attitudes, knowledge, and practices were characterized using descriptive statistics. The primary dependent outcome variables were eye health practices and knowledge (measured as having heard of the disease and correctly identifying risk factors). The American Academy of Ophthalmology (AAO) recommends that, even in the absence of signs or risk factors for eye disease, adults should receive at least one comprehensive eye examination by age 40, and have regular eye examinations every 2-to-4 years between the ages of 40 and 54 years, every 1-to-3 years between the ages of 55 and 64 years, and every 1 to 2 years after 64 years old. We considered optimal eye care as having received a dilated eye examination and having had an eye exam in the past year.

Predictive variables assessed for relationships with the outcomes included having a primary care provider (PCP), having health insurance, health eye insurance, age group, immigrant status, educational attainment, socioeconomic status (SES, income category), and acculturation score (ASASFA). Bivariate analyses were conducted using chi-squared for categorical variables and Student’s *t*-test for continuous variables through STATA software version 16.0. Predictor variables which held statistical significance in binary analyses, as well as variables which held face validity, were included in a multivariable logistic regression model that examined factors associated with having an eye examination within the last year. Statistical significance was defined as a p-value ≤ 0.05.

The University of California, San Francisco’s Human Research Protection Program and University of California, Berkeley’s Office for the Protection of Human Subjects reviewed and deemed this study as exempt human subjects research.

## Results

### Demographics

Of 340 responses collected, 256 completed surveys were eligible, with high overall survey completion rates. The top SF Bay Area county represented was SF (50.4%). A majority of the respondents were 40–54 years old (85.6%), male (76.6%), high school graduates (99.6%) and employed (60.5%), with a household income between $50,000 and $99,999 (65.5%). In our study population, 84.2% reported having a PCP, 85.6% had vision insurance, and 95.7% had some form of health insurance. Additionally, 29.1% were Philippine-born, and of those born in the Philippines, 63.8% immigrated more than 20 years ago. The mean acculturation ASASFA score was 32.5. Four of 256 respondents took the survey in Tagalog; the rest of the respondents used English. Only six phone surveys were conducted.

### Knowledge of Eye Diseases

**Fig. 2:**
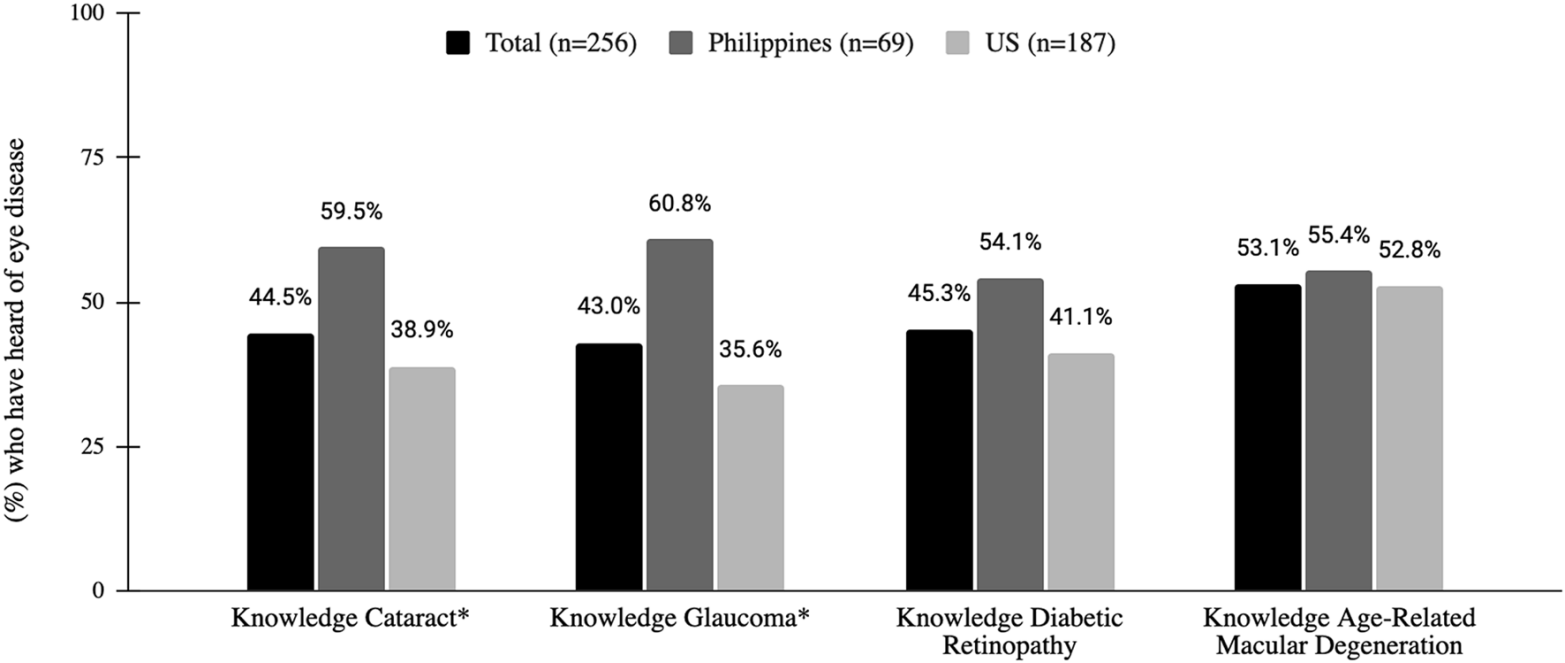
Respondents’ eye diseases knowledge of those born in the Philippines vs. born in the U.S. *P-value < 0.05 comparing U.S. and Philippines born

Knowledge about different eye diseases varied. In our study population, 44.5% were aware of cataracts, 43.0% glaucoma, 45.3% DR, and 53.1% age-related macular degeneration (ARMD). Those who were less aware about eye diseases were those who were born in the U.S. (e.g. 35.6% U.S. vs. 60.8% Philippines aware of glaucoma; p < 0.05; see Fig. 2) and those between 40–54 years old (e.g. 59.4% unaware of cataracts, 58.0% unaware of diabetic retinopathy, 60.7% unaware of glaucoma; p < 0.05). More than half of survey respondents understood that diabetes and hypertension are risk factors for eye diseases (63.1% and 63.7%, respectively) while less than half of survey respondents knew that smoking is associated with greater risk for eye disease (36.4%) (Table 1).

**Table 1:** Eye disease knowledge and attitudes by birthplace

	Total (n = 256, %; somewhat or strongly agree)	Born in the Philippines (%)	Born in the United States (%)	p-value
Diabetes is associated with greater risk of eye disease	63.1	71.2	59.4	0.10*
Hypertension is associated with greater risk of eye disease	63.7	81.1	56.7	0.00*
Smoking is associated with greater risk of eye disease	36.4	63.5	24.9	0.00*
Good overall health is important for good eye health	42.0	63.5	32.2	0.00*
Good eye health is important for good overall health	56.0	69.9	50.6	0.00*

Bold values highlight the statistically significant numbers

*p-value < *0.05*

### Attitudes about Vision and Eye Health

**Fig. 3:**
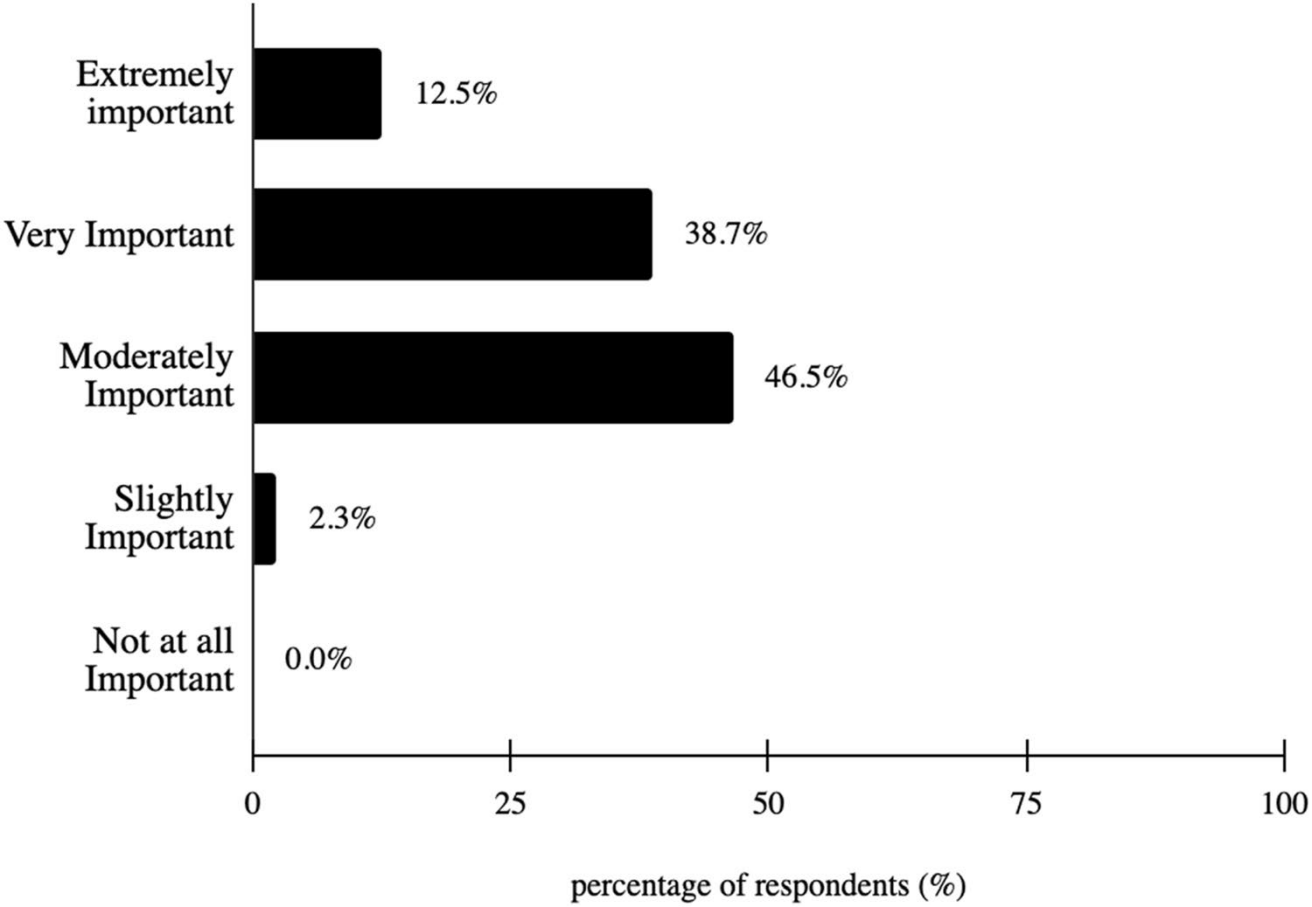
How important is it to you that you have an eye doctor?

**Fig. 4:**
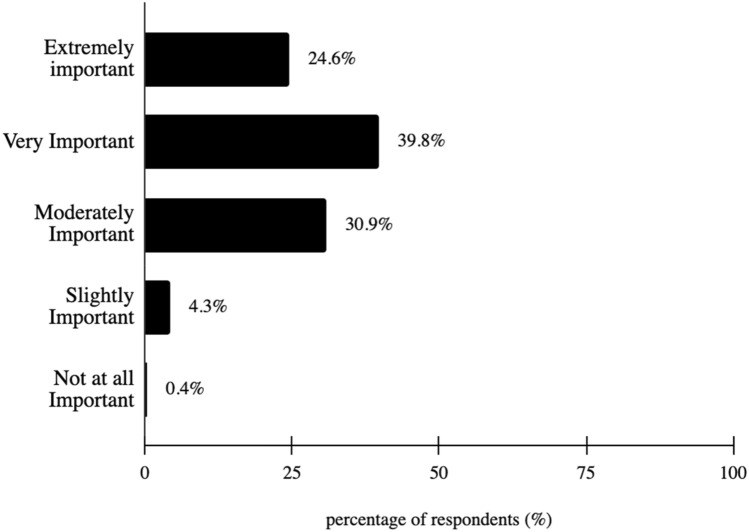
How important is vision to you?

**Fig. 5:**
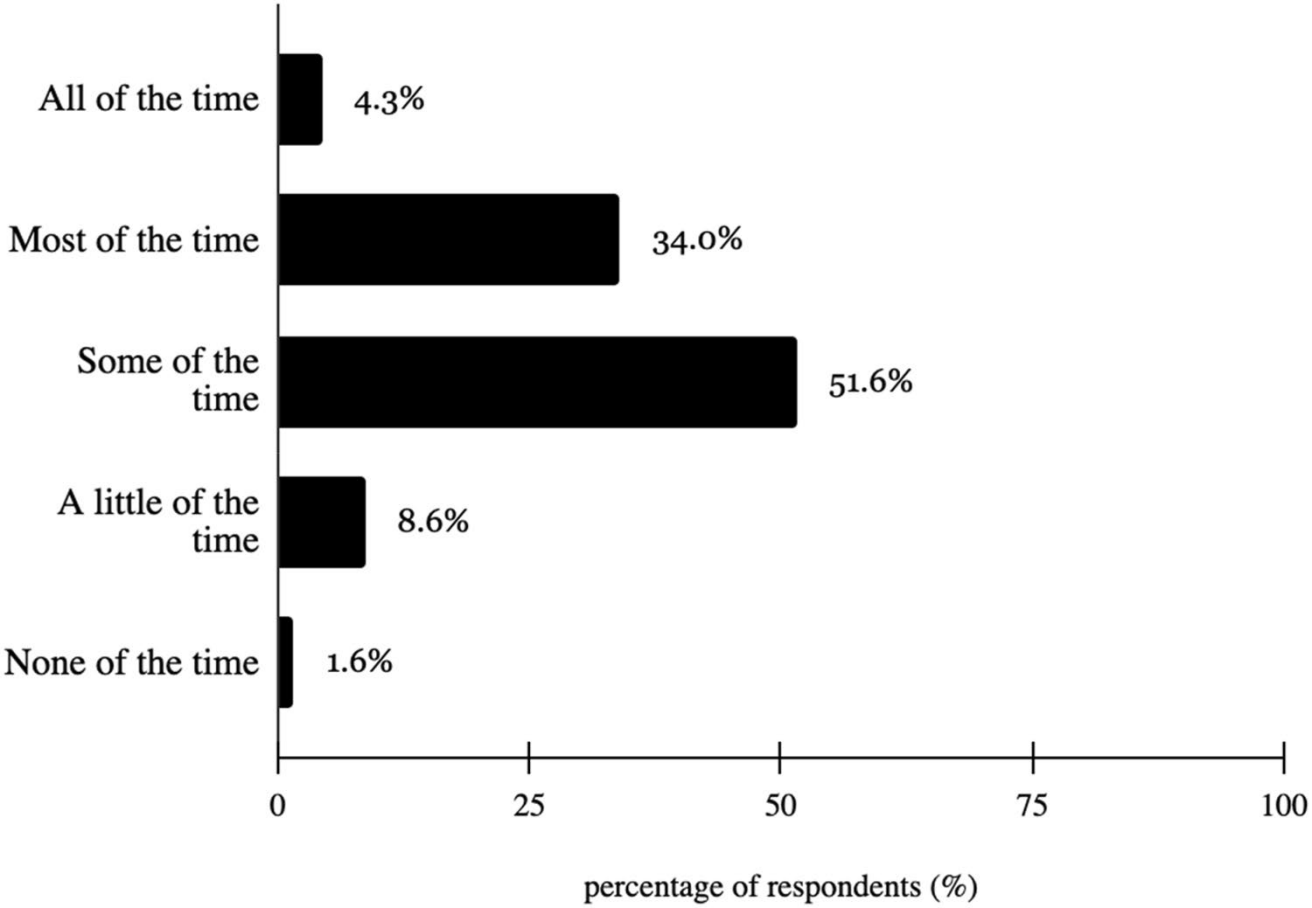
How much of the time do you worry about your eyesight?

A majority of survey respondents (95.3%) stated vision is extremely, very, or moderately important to them, with a modal response of “moderately important” at 46.5% (Fig. 3). Additionally, 97.7% of survey respondents stated having an eye doctor is extremely, very or moderately important, with a modal response of “very important” at 39.8% (Fig. 4). Lastly, 89.8% of survey respondents worry about vision all, most, or some of the time, with a modal response of “some of the time” at 51.6% (Fig. 5).

### Practices concerning Eye Diseases

In our study population, 81.6% received an eye exam ≤ 1 year and 92.5% had a dilated eye examination. Those less likely to have had an eye exam ≤ 1 year in our study population were those who had no PCP (13.1%), no eye insurance (14.4%), and who immigrated less than 20 years ago from the Philippines to the United States (Table 2). Those less likely to ever have had their eyes dilated (p < 0.05) were those who were: lower SES, had no vision insurance, had no health insurance, had no PCP, immigrated after 2000, and had a higher acculturation score (Table 3). After adjusting for age, sex, eye insurance, and presence of self-reported diabetes or hypertension, having a current PCP resulted in a 2.6-fold increased odds of having had an eye exam within the last year (Table 4).

**Table 2:** Demographic characteristics of survey respondents by recent eye exam

	Total	Received an Eye Exam ≤ 1 year	Did not receive an Eye Exam ≤ 1 year	p-value
Gender (n = 256, %)				0.64
Female	23.4	22.8	26.1	
Male	76.6	77.2	73.9	
Age (n = 256, years, %)				0.28
40–54	85.6	86.1	82.1	
55–64	12.1	11.0	17.0	
65 +	2.3	2.9	0.0	
Has PCP (n = 256, %)				0.00*
No	13.1	10.0	26.7	
Yes	86.9	90.0	73.3	
County (n = 256, %)				0.81
Alameda	11.3	11.0	12.8	
Contra Costa	4.7	4.8	4.3	
Marin	3.5	4.3	0.0	
Napa	5.5	4.8	8.5	
San Francisco	50.4	51.7	44.7	
San Mateo	9.4	8.6	12.8	
Santa Clara	8.6	8.1	10.6	
Solano	4.7	4.8	4.3	
Sonoma	2.0	1.9	2.1	
Immigration status (n = 255, %)				0.64
Born in the U.S	70.6	71.5	68.1	
Philippines (n = 69)	29.1	28.5	31.9	0.01*
* i. Immigrated from PI* ≤ *20 years ago*	36.2	29.3	72.7	
* ii. Immigrated from PI* > *20 years ago*	63.8	70.7	27.3	
Acculturation (n = 256, mean)				0.07
Acculturation Score	32.5	32.8	31.1	
Employment status (n = 253, %)				0.45
Employed full-time	60.5	63.0	59.9	
Employed part-time	2.4	2.9	0.0	
Unemployed	15.8	15.0	19.6	
Self-employed	7.9	7.3	10.9	
Disabled	4.7	5.8	0.0	
Military	0.8	0.97	0.0	
Retired	7.9	8.2	6.5	
Education (n = 253, %)				0.70
None completed	0	0.0	0.0	
Some high school	0.4	0.5	0.0	
Graduated high school	12.3	11.2	17.0	
Trade/Tech/Vocational Training	25.3	25.2	25.5	
Associate’s degree	32.0	34.0	23.4	
Some college, less than 4 years	14.6	13.6	19.2	
Bachelor’s degree or equivalent	11.9	11.7	12.8	
Graduate school started or completed	3.6	3.9	2.1	
Total household income (n = 249, %)				0.70
Less than $50,000	12.9	11.9	17.0	
$50,000 to $99,999	65.5	66.8	59.6	
$100,000 to $149,999	14.5	13.9	17.0	
Greater than $150,000	7.2	7.4	6.4	
Eye insurance (n = 256, %)				0.03*
Not insured	14.4	12.1	25.0	
Insured	85.6	87.9	75.0	
Health insurance status (n = 256, %)				0.44
Not insured	4.3	96.2	93.6	
Insured	95.7	3.8	6.4	
* i. Medicare*	35.2	–	–	
*ii. Medicaid/Medi-Cal*	39.5	–	–	
* iii. Other*	25.3	–	–	
Self reported health diagnoses (n = 256, %)				
Diabetes	12.5	–	–	
Hypertension	51.2	–	–	
Stroke	14.8	–	–	

Bold values highlight the statistically significant numbers

*p-value < *0.05*

**Table 3:** Factors that affect receipt of eye dilation exam

	Have received an eye dilation exam	Never received an eye dilation exam (n = 19)	p-value
Gender (%)			0.04*
Female	21.7	42.1	
Male	78.3	57.9	
Age (n = 253, year, %)			0.04*
40–54	85.5	84.2	
55–64	12.8	5.3	
65 +	1.7	10.5	
PCP (n = 243, ta%)			0.00*
No	11.0	43.8	
Yes	89.0	56.3	
Immigration status (n = 251, %)			0.18
Born in the U.S	72.4	57.9	
Born in the Philippines (n = 67/255)	27.6	42.1	0.00*
* i. Immigrated from PI* ≤ *20 years ago*	27.1	87.5	
* ii. Immigrated from PI* > *20 years ago*	72.9	12.5	
Acculturation score (mean)	33	28	0.00*
Household income (n = 246, %)			0.04*
Less than $50,000	11.5	31.6	
$50,000 to $99,999	65.6	63.2	
$100,000 to $149,999	15.0	5.26	
Greater than $150,000	7.9	0	
Eye insurance (%)			0.02*
Not Insured	13.0	33.3	
Insured	87.0	66.7	
Health insurance (n = 253, %)			0.01*
Not Insured	3.42	15.8	
Insured	96.6	84.2	
* i. Medicare (n* = *87)*	32.5	57.9	–
*ii. Medicaid/Medi-Cal (n* = *101)*	41.9	15.8	-

Bold values highlight the statistically significant numbers

*p-value < *0.05*

**Table 4:** Factors associated with having an eye examination within the last year

Variable	Odds Ratio (95% CI)	p-value
Age ≥ 55	0.93 (0.31–2.84)	0.9
Male sex	1.31 (0.58–2.96)	0.51
Having a PCP	2.60 (1.01–6.64)	0.047*
Having eye insurance	2.08 (0.79–5.49)	0.14
Self-reported diabetes	0.59 (0.20–1.76)	0.34
Self-reported hypertension	0.98 (0.47–2.04)	0.96

Bold values highlight the statistically significant numbers

*p-value < *0.05*

### Eye Examinations and the Effect of COVID-19

When asked about future eye check appointments, several online survey respondents report that COVID-19 has affected their ability to get their eye examinations, “avoiding clinics during COVID” or are “[fearful] of COVID”.

## Discussion

To our knowledge, this is the first study focusing on the facilitators, practices, and needs of Filipino-Americans in Northern California with respect to eye care and eye health. Our data suggest that a large proportion of older Filipino-American adults within our study population are receiving adequate eye care as recommended by the AAO [24], with 81.6% having had an eye exam ≤ 1 year, and 91.5% ever having a dilated eye examination. These are higher rates of eye care receipt compared to Chinese-, Latino-, and Black-American data [11, 13, 25]. Several factors may explain why a large proportion of study participants met standards. First, most respondents completed the survey online, which may suggest higher health literacy and economic status; this might extend to their ability to schedule, attend, and prioritize vision appointments. Second, vision was important for a majority of the survey takers (95.3%), suggesting participation bias. Third, our study sample was a highly insured population (95.7% insured and 85.6% vision insured), similar to studies showing that a large proportion of Filipino-Americans have health insurance [26, 27]. However, having health insurance does not guarantee access to eye care, which may be covered separately. Of those who reported never having a dilated eye examination, only 84.2% had health insurance coverage. Additionally, we found that the categories of low SES, immigrants before 2000, higher acculturation scores, and 65 + years old, as well as those without vision insurance, health insurance, and a PCP were significantly associated with not having a dilated eye examination. These findings align with studies showing how those in poverty and without vision insurance have delayed and the least access to appropriate eye care [28, 29].

Despite a large proportion of our study receiving adequate eye vision screenings, there was a significant gap in eye disease and health knowledge. In general, only around half of our study participants were aware of different eye diseases. This is striking compared to a 2005 public KAP survey, in which 90.0% were aware of glaucoma (vs. 43.0% in our study population), and 51.0% were aware of DR (vs. 45.3% in our study population) [20]. DR has established recommendations for follow-up, treatment and disease management. Furthermore, diabetes is more prevalent in the Filipino-American population compared with other Asian-American and some non Asian-American groups [14, 15]. With a high proportion of our study population with self-reported diabetes (12.5%) and PCP access (86.9%), alongside clear existing U.S. guidelines for regular DR screening [30], one would assume that PCPs are more likely to teach and inform patients about DR. However, we found only 63.1% were aware that diabetes is strongly associated with greater risk of eye disease. Although our study was not designed to determine the exact reasons for lack of eye knowledge, we postulate there could be an issue of communication between the physician and the patient, that ties into cultural humility and teach-back methods. A significant gap in knowledge was also found in our study between U.S.-born Filipino-Americans and Philippine-born Filipino-Americans. U.S.-born Filipino-Americans were less knowledgeable about eye health versus Philippine-born Filipino-Americans. Filipino-Americans have a large diaspora and patient education materials may not be accessible or given at appropriate levels of health literacy. This may be supported given findings that a higher mean acculturation score was found in the group that had a dilated eye examination versus those that never had a dilated eye examination (33 vs. 28). Alternatively, it may be attributed to differences in cultural norms pronounced in immigrant populations. Discussions concerning diseases and effective remedies are central to Filipino social life [31], which can possibly explain the knowledge discrepancies observed.

We found high rates of eye care screening in this largely insured study population. This might imply that eye health goals were met for the Filipino-American community. However, juxtaposing the current high screening rates with the overall poor knowledge around eye diseases and conditions, something remains amiss. It suggests a potential lack of agency patients may experience concerning their care in which they may be blindly following clinician recommendations, lacking knowledge to make an informed decision. Without recognizing the full impact of eye diseases, this may diminish the significance of vision screenings and follow-up care for vision maintenance. Drop offs in care and follow-up appointments may happen. It is a clinician’s prerogative to advocate for patients beyond just reaching numerical benchmarks so that patients can make informed choices, empowering them to take charge of their health.

Previous work suggests that a more integrated and collaborative approach is necessary for patient agency in decision-making. For example, a review by Domingo et al. on various cardiovascular and diabetes management interventions emphasized the importance of incorporating Filipino values, community, and family members to ensure the improvement in outcomes [32, 33]. These learnings show how culturally sensitive programs and an ethnically concordant team of care providers can promote eye health and prevent eye diseases in the Filipino-American community.

Currently, routine eye care services such as regular eye exams are excluded from Medicare coverage [34]. While our study did not specifically look into this issue, we found that more participants had health insurance than had vision insurance. This indicates a push for health policy efforts to fold vision insurance into health insurance instead of the current carve-out plans. Policy needs to be informed by accurate and up-to-date data, but there is little and outdated literature focused on Filipino-American population needs. Asian-American data is often aggregated, and this data aggregation hides issues that are uniquely faced by a given population, especially one which itself has a rich diversity. In the Philippine diaspora alone, 256 languages are spoken across almost 2000 inhabited Philippine islands [35–37]. Disaggregating up-to-date Asian-American data is necessary to understand how to better serve specific communities within this nonrepresentative monolith data point. This dual approach will ensure more people are receiving adequate and needed health care, including vision care.

It is important to be proactive, not reactive, towards vision loss. High-quality educational outreach that is accessible, culturally aligned, and clearly communicated [33, 38–40] will underpin the population’s belief that vision is important. Implementation of culturally sensitive strategies through health fairs is one way health education can be achieved in the older adult Filipino-American population [38], especially for those with difficulty getting to appointments; this is particularly poignant during the COVID-19 era of social distancing. A majority of our study participants have a primary care provider, which may suggest a benefit of better integration of eye care in primary care. Lastly, access and utilization of language interpreters by Filipino-Americans, especially when navigating different specialties within the healthcare system, should be assessed. Future vision health research must test the effectiveness of these interventions in the Filipino-American community. More qualitative studies are needed to tease out nuances and elucidate how care can have a more culturally humble and sensitive lens.

## Limitations

This quantitative research was conducted during the COVID-19 pandemic shelter-in-place mandate. Self-reporting has its limitations. The research team shared this survey amongst organizations that serve Filipino-American populations; however, this anonymous survey could not 100% guarantee that all survey takers were part of the inclusion group. While two different survey modalities were offered to capture different demographics, our survey takers may not be fully representative of the larger Bay Area Filipino-American community. The study population was slightly different from the Filipino-American population of the Bay Area compared to the 2019 U.S. Census. The 2019 U.S. Census covered Filipino-American data in six of nine Bay Area counties, reporting that 96% had health insurance and 94% was a high school graduate or higher, which is similar to our study population. The report also showed a higher female to male ratio, average household income ($134,000), and number of foreign born Filipino-Americans (50%) [26]**.** We employed a convenience sample which may have affected the generalizability of our population sample and introduced selection bias. Future research should replicate data post-pandemic to compare and contrast data outcomes.

## Conclusion

In our study, we found high rates of eye care screening in this largely insured sample population, but found disparities in eye care associated with low SES, recency in immigration, and lack of health and vision insurance. A significant gap in knowledge about eye diseases was also found in this study population, suggesting a lack of and need for educational outreach in this community. Our next steps are to advocate for a culturally sensitive and patient-empowering approach when discussing vision health. Additionally, conducting further qualitative studies can complement our KAP survey, allowing further exploration in understanding the nuances, experiences, and needs of Filipino-Americans in terms of eye care.

## Supplementary Information

Below is the link to the electronic supplementary material.

Supplementary file1 (DOCX 39 kb)
