# Supplementary material for: Ang Ating Mata: Disparities in Eye Health Knowledge, Attitudes and Practices among Older Adult Filipino-Americans in the San Francisco Bay Area Counties
Source: J Immigr Minor Health. 2022 Jun 29;25(1):104–14. doi: 10.1007/s10903-022-01371-3 (PMC9244114; doi:10.1007/s10903-022-01371-3)
Supplement: Supplementary file 1 — Supplementary file1 (DOCX 39 kb) [file 10903_2022_1371_MOESM1_ESM.docx]

**Appendix 1: English KAP Survey**

**Introduction to KAP Survey:**

Researchers at the University of California San Francisco are gathering information about eye health in Filipino Americans aged 40 and older living in the San Francisco Bay Area. This online survey will ask you questions about your knowledge, attitudes, and practices related to eye diseases.

Your participation in the study is voluntary. Your name, address, or other personal information that can identify you will not be used for data collection. Any information you provide will be confidential. The questionnaire takes about 10-15 minutes to complete.

You will receive a $5 gift card for your time. To receive your gift card, please provide your email at the end of the survey. Again, your personal information will not be used for data collection.

If you have any questions about this survey, please contact the study research team at 510-XXX-XXXX and leave a voicemail, or send us an email at XXXXX@gmail.com.

Thank you/Maraming Salamat!

**Eligibility Questions:**

1. Do you self-identify as a Filipino American?
   1. Yes
   2. No
2. How old are you?
   1. 18-39
   2. 40-54
   3. 55-64
   4. 65+
3. What county in the Bay Area do you live in?
   1. Alameda
   2. Contra Costa
   3. Marin
   4. Napa
   5. San Francisco
   6. San Mateo
   7. Santa Clara
   8. Solano
   9. Sonoma
   10. Other
4. Select all that apply. What languages did you hear growing up in your family?
   1. Tagalog
   2. English
   3. Hiligaynon
   4. Cebuano
   5. Kapampangan
   6. Ilocano
   7. Other filipino language/dialect: ______

**KNOWLEDGE, ATTITUDES, AND PRACTICES (KAP) SURVEY**

1. In general, would you say your health is:
   1. Excellent
   2. Very good
   3. Good
   4. Fair
   5. Poor
2. Would you say your eyesight in general (without contacts or glasses) is:
   1. Excellent
   2. Very good
   3. Good
   4. Fair
   5. Poor
3. Do you wear...
   1. Prescription Glasses
   2. Contact lenses
   3. Both prescription glasses and contact lenses
   4. Reading glasses only
   5. None of the above
4. At the present time, would you say your eyesight using both eyes (with glasses or contact lenses, if you wear them) is:
   1. Excellent
   2. Very good
   3. Good
   4. Fair
   5. Poor

**Knowledge of eye diseases**

1. Do you agree or disagree that diabetes is associated with greater risk of eye disease?
   1. Strongly agree
   2. Somewhat agree
   3. Somewhat disagree
   4. Strongly disagree
   5. Not sure
2. Do you agree or disagree that hypertension, or high blood pressure, is associated with greater risk of eye disease?
   1. Strongly agree
   2. Somewhat agree
   3. Somewhat disagree
   4. Strongly disagree
   5. Not sure
3. Do you agree or disagree that smoking is associated with greater risk of eye disease?
   1. Strongly agree
   2. Somewhat agree
   3. Somewhat disagree
   4. Strongly disagree
   5. Not sure
4. Select all that apply. Have you ever heard of the following?
   1. Cataract—a clouding of the lens of the eye
   2. Diabetic retinopathy or diabetic eye disease—a complication of diabetes that affects your vision
   3. Glaucoma—a disease that damages the optic nerve and causes loss of side or peripheral vision
   4. Age-related macular degeneration—an eye disease that affects older people and causes a loss of central or straight-ahead vision
   5. Myopia—trouble seeing things that are far away
   6. Hyperopia—trouble seeing things that are close up
   7. Presbyopia—the inability to focus up close often referred to as the aging eye condition
   8. Any other eye condition or eye disease? (SPECIFY)
   9. None of the above

**Attitudes**

1. Do you agree or disagree with the following statement: “Good eye health is important to overall health.”
   1. Strongly agree
   2. Somewhat agree
   3. Neither agree nor disagree
   4. Somewhat disagree
   5. Strongly disagree
2. Do you agree or disagree with the following statement: “Good overall health is important to good eye health.”
   1. Strongly agree
   2. Somewhat agree
   3. Neither agree nor disagree
   4. Somewhat disagree
   5. Strongly disagree
3. How important is it to you that you have an eye doctor?
   1. Extremely important
   2. Very important
   3. Moderately important
   4. Slightly important
   5. Not at all important
4. How important is vision to you?
   1. Extremely important
   2. Very important
   3. Moderately important
   4. Slightly important
   5. Not at all important
5. How much of the time do you worry about your eyesight?
   1. All of the time
   2. Most of the time
   3. Some of the time
   4. A little of the time
   5. None of the time

**Prevention and treatment practices**

1. About how long ago was the last time you saw or visited a health care provider? (A health care provider is a doctor, a nurse practitioner, or any other medical professional who has given you a physical examination or written prescriptions for you)
   1. Within the past month (anytime less than 1 month ago)
   2. Within the past year (1 month but less than 12 months ago)
   3. Within the past 2 years (1 year but less than 2 years ago)
   4. 2 or more years ago
   5. Never
2. Do you have a primary care provider?
   1. Yes
   2. No
   3. Unsure
3. When was the last eye examination you had by an eye care provider such as an ophthalmologist or optometrist?
   1. Within the past month (anytime less than 1 month ago)
   2. Within the past year (1 month but less than 12 months ago)
   3. Within the past 2 years (1 year but less than 2 years ago)
   4. 2 or more years ago
   5. Never
4. *(if answered a,b,c,d for 2)* What was the one MAIN reason you had your eyes examined the last time by an eye care provider? (OPEN)
5. *(if answered e for 2)* Is there a particular or **one MAIN** reason why you have never had your eyes examined by an eye care provider? **(OPEN)**
6. Have you had any dilated eye examination—that is, drops would have been placed in your eyes, and might have caused blurry vision or sensitivity to light—in the past 12 months
   1. Yes
   2. No
7. *(if b for 5)* Have you ever had a dilated eye examination—that is, drops would have been placed in your eyes, and might have caused blurry vision or sensitivity to light?
   1. Yes
   2. No
8. Select all that apply. What health insurance do you have?
   1. No insurance
   2. Medicare
   3. Medicaid (Medi-Cal)
   4. Other government provided coverage (VA, Tricare, etc)
   5. My insurance is offered through an employer
   6. My insurance is purchased privately
   7. My insurance is purchased through an exchange
   8. Not sure
9. Do you have insurance coverage for routine eye exams or glasses?
   1. Yes
   2. No
   3. Unsure
10. Select which statement best describes how your insurance situation impacts how often you have your eyes checked. 'Because of my insurance situation...'
    1. I have eye exams less frequently than I would like
    2. I never have eye exams because I don't have insurance coverage
    3. I am able to have my eyes checked as often as I would like
    4. My insurance situation doesn't affect how often I have eye exams because I pay for my eyecare out of pocket
11. Select all that apply. Has the doctor ever told you that…
    1. you had diabetes?
    2. you had high blood pressure/hypertension?
    3. had a stroke?
    4. diabetes has affected blood vessels in your eyes or that you had diabetic retinopathy or diabetic eye disease?
    5. you had macular degeneration (AMD) or senile macular degeneration, which is damage of the macular in the back part of your eye, of the retina?
    6. you had cataracts in either eye?
    7. you had glaucoma in either eye?
    8. you had ptosis, which is low-lying or drooping upper eyelid(s)?
12. *(if a for 10)* Are you currently taking pills for high blood sugar?
    1. Yes
    2. No
13. *(if a for 10)* Do you use insulin for high blood sugar?
    1. Yes
    2. No
14. *(If b for 10)* Are you currently taking pills for high blood pressure?
    1. Yes
    2. No
15. How often do you currently smoke tobacco?
    1. Daily
    2. Less than daily
    3. Not at all
16. *(if b for 14)* In the past, have you ever smoked tobacco on a daily basis?
    1. Yes
    2. No
17. *(if c for 14)* In the past, did you smoke tobacco…
    1. on a daily basis
    2. less than daily
    3. not at all

**Demographic questions**

1. Gender
   1. Male
   2. Female
   3. Other______________
2. What is your current employment status?
   1. Full-time employment. Occupation: ______________
   2. Part-time employment. Occupation: ______________
   3. Unemployed
   4. Self-employed
   5. Disabled
   6. Student
   7. Military
   8. Retired
3. Where were you born?
   1. In Philippines
   2. In the United States
   3. Outside of the United States and not in the Philippines
4. Education
   1. No schooling completed
   2. Some high school (no diploma) or less
   3. High school diploma
   4. Trade/technical/vocational training
   5. Associate's degree
   6. Some college, but less than a 4-year degree
   7. Bachelor's degree or equivalent completed
   8. Graduate school started or completed
5. Total Household Income
   1. Less than $25,000
   2. $25,000 to $49,999
   3. $50,000 to $74,999
   4. $75,000 to $99,999
   5. $100,000 to $124,999
   6. $125,000 to $149,999
   7. $150,000 to $174,999
   8. $175,000 to $199,999
   9. $200,000 or more
6. Where should we send you your gift card? (please write your email address)
7. Select all that apply. Can we contact you in the future for further participation in research?
   1. Yes (email)
   2. Yes (phone number)
   3. No, I do not want to help future research.

For the following statements, please choose the answer most applicable to you.
If you are taking survey on mobile phone: please click on each statement or turn your phone to the side to see response options.

**Options for the following statements were: Only Philippine languages, More Philippine languages than English, Both equally, More English than Philippine languages, and Only English*

1. Language(s) spoken at home
2. Language(s) read and spoken
3. Language(s) spoken with friends
4. Language(s) of preferred movies, TV, and media
5. Language(s) used with thinking
6. Language(s) spoken as a child
7. Language(s) of TV programs usually watched

**Options for the following statements were:* All Filipinos, More Filipinos than Americans, about half and half, more Americans than Filipinos, All Americans

1. Ethnicity of visitors or persons visited
2. Ethnicity of close friends
3. Ethnicity of social gatherings
4. Ethnicity of children's friends (if not applicable, please leave blank)

**Tagalog KAP Survey**

**Panimula sa KAP Survey:**

Ito ay isang pananaliksik o pagsusuri ng Cocohoba Lab sa UCSF (Unibersidad ng California, San Francisco) na ang layunin ay magtipon ng impormasyon tungkol sa kalusugan ng mata ng mga Filipino-American na may edad na 40 o pataas sa Bay Area. Ang mga sumusunod na katanungan ay tungkol sa iyong mga kaalaman, saloobin at kasanayan kaugnay ang kalusugan ng mata at mga sakit sa mata.

Ang inyong partisipasyon sa pagsusuri na ito ay kusang-loob at nasa sa inyong sariling kagustuhan. Ang inyong pangalan, adres, at ibang personal na impormasyon na maaring magamit para kayo ay matukoy ay hindi gagamitin sa koleksyon ng data. Ang lahat ng impormasyon na inyong ihahayag sa amin ay mananaliting kumpidensyal.

Ang pag kumpleto ng survey ay tatagal ng mga 10-15 minuto. Para sa mga katanungan tungkol sa survey at pagsusuri na ito, tumawag sa 510-972-4580 at magiwan ng voicemail o magpadala ng mensahe sa email [eyehealthstudyUCSF@gmail.com](mailto:eyehealthstudyUCSF@gmail.com)

Maraming Salamat po!

**Mga tanong tungkol sa mga katangian na angkop sa survey**

1. Kinikilala mo ba ang iyong sarili bilang isang Filipino o Filipino-American?
   1. Oo
   2. Hindi
2. Ilang taon ka na?
   1. 18-39
   2. 40-54
   3. 55-64
   4. 65+
3. Alin sa mga sumusunod na county sa Bay Area ka nakatira?
   1. Alameda
   2. Contra Costa
   3. Marin
   4. Napa
   5. San Francisco
   6. San Mateo
   7. Santa Clara
   8. Solano
   9. Sonoma
   10. Ibang county
4. Piliin ang lahat ng naaangkop. Alin sa mga sumusunod na wika ang iyong kinalakihan na naririnig sa inyong pamilya?
   1. Tagalog
   2. English
   3. Hiligaynon
   4. Cebuano
   5. Kapampangan
   6. Ilocano
   7. Ibang wikang Filipino: ______

**KAALAMAN, SALOOBIN, AT KASANAYAN SURVEY**

1. Ano ang iyong saloobin sa kalagayan ng iyong pangkalahatan na kalusugan :
   1. Sobrang mabuti
   2. Napakabuti
   3. Mabuti
   4. Sapat lang
   5. Mahina na
2. Ano ang iyong saloobin sa kalagayan ng kalusugan ng iyong mga mata?
   1. Sobrang mabuti
   2. Napakabuti
   3. Mabuti
   4. Sapat lang
   5. Mahina na
3. Nagsusuot ka ba ng …
   1. Salamin
   2. Contact lenses
   3. Salamin at contact lenses
   4. Salamin para sa pagbabasa (reading glasses)
   5. Wala sa itaas ang nalalpat
4. Sa kasalukuyan, ano ang iyong saloobin tungkol sa iyong paningin sa dalawang mata (gamit ang salamin o contact lens kung ikaw ay nagsusuot nito)
   1. Sobrang mabuti
   2. Napakabuti
   3. Mabuti
   4. Sapat lang
   5. Mahina na

**Kaalaman tungkol sa mga sakit sa mata**

1. Ikaw ba ay sumasang-ayon o hindi sumasang-ayon na ang diyabetis (diabetes) ay nauugnay sa mas malaking panganib ng pagkakaroon ng sakit sa mata?
   1. Matinding sumasang-ayon
   2. Sumasang-ayon
   3. Hindi sumasang-ayon
   4. Matinding hindi sumasang-ayon
   5. Hindi sigurado
2. Ikaw ba ay sumasang-ayon o hindi sumasang-ayon na ang hypertension o pagtaas ng presyon ay nauugnay sa mas malaking panganib ng pagkakaroon ng sakit sa mata?
   1. Matinding sumasang-ayon
   2. Sumasang-ayon
   3. Hindi sumasang-ayon
   4. Matinding hindi sumasang-ayon
   5. Hindi sigurado
3. Ikaw ba ay sumasang-ayon o hindi sumasang-ayon na ang paninigarilyo ay nauugnay sa mas malaking pagkakataon ng pagkakaroon ng sakit sa mata?
   1. Matinding sumasang-ayon
   2. Sumasang-ayon
   3. Somewhat disagree
   4. Strongly disagree
   5. Hindi sigurado
4. Piliin ang lahat ng naaangkop. Alin sa mga sumusunod na sakit sa mata ay meron ka ng kaalaman o dati niyo ng narinig?
   1. Katarata—pag-ulap o pag labo ng lens ng mata
   2. Diabetic retinopathy o sakit sa mata ng diyabetik— isang komplikasyon ng diyabetis sa mata na nakakaapekto sa paningin
   3. Glaucoma— sakit na nakakasira sa ugat ng mata at nakakawala ng paningin sa gilid ng mata
   4. Macular Degeneration na may kaugnayan sa edad—sakit sa mata na nakakaapekto sa mga mas nakakatanda at maaring magdulot ng pagkawala ng sentral o diretsong paningin
   5. Myopia—mahinang paningin sa malayo
   6. Hyperopia—mahinang paningin sa malapit
   7. Presbyopia— paglabo ng mata dahil sa kawalan ng kakayahan na mag-focus; ito ay tinatawag din na kondisyon ng pagtanda ng mata o ‘aging eye condition’
   8. Iba pang kondisyon o sakit sa mata? (Tukuyin o ilarawan)
   9. Hindi alam o hindi sigurado

**Mga saloobin**

1. Ikaw ba ay sumasang-ayon o hindi sumasang-ayon sa sumusunod: “Ang mabuting kalusugan ng mata ay importante sa kalusugan ng buong pangangatawan.”
   1. Matinding sumasang-ayon
   2. Sumasang-ayon
   3. Ni sumasang-ayon o hindi sumasang-ayon
   4. Hindi sumasang-ayon
   5. Matinding hindi sumasang-ayon
2. Ikaw ba ay sumasang-ayon o hindi sumasang-ayon sa sumusunod: “Ang mabuting kalusugan ng buong pangangatawan ay importante sa kalusugan ng mata.”
   1. Matinding sumasang-ayon
   2. Sumasang-ayon
   3. Ni sumasang-ayon o hindi sumasang-ayon
   4. Hindi sumasang-ayon
   5. Matinding hindi sumasang-ayon
3. Gaano kaimportante sa iyo na meron kang doktor para sa mata?
   1. Sobrang napakaimportante
   2. Napakaimportante
   3. Medyo Importante
   4. Hindi masyadong importante
   5. Talagang hindi importante
4. Gaano kaimportante sa iyo ang iyong paningin?
   1. Sobrang napakaimportante
   2. Napakaimportante
   3. Medyo Importante
   4. Hindi masyadong importante
   5. Talagang hindi importante
5. Gaano ka kadalas mag-alala tungkol sa iyong paningin?
   1. Sa lahat ng oras
   2. Madalas
   3. Paminsan-minsan
   4. Madalang
   5. Hindi ako nag-aalala

**Prevention and treatment practices**

1. Kailan ang huling beses na kayo ay bumisita sa iyong tagabigay ng pangangalaga sa kalusugan? (Ang tagabigay ng pangangalaga sa kalusugan ay ang iyong doktor, nurse practitioner (NP), o ibang propesyonal na pang medikal na nagbibigay ng eksaminasyong pisikal o ng receta para sa iyong gamot).
   1. Sa loob ng nakaraan na buwan
   2. Sa loob ng nakaraan na taon
   3. Sa loob ng nakaraan na 1 taon
   4. Higit sa 2 taon na ang nakalipas
   5. Hindi kailanman
2. Meron ka bang pinupuntahan na regular na doktor (para magpacheck-up sa iyong kalusugan)?
   1. Oo
   2. Wala
   3. Hindi sigurado
3. Kailan ang huling beses na kayo ay nagpacheck-up ng mata sa doktor ng mata (ophthalmologist o optometrist)
   1. Sa loob ng nakaraan na 30 araw
   2. Sa loob ng nakaraan na 1 taon
   3. Sa loob ng nakaraan na 2 taon
   4. Higit sa 2 taon na ang nakalipas
   5. Hindi kailanman
4. *(if answered a,b,c,d for 2)* Ano ang isang pangunahing dahilan ng iyong pag-papacheck-up ng mata mula sa doktor ng mata? (OPEN)
5. *(if answered e for 2)* Ano ang **isang pangunahing dahilan** kung bakit hindi ka pa nagpapacheck-up ng mata? (OPEN)
6. Nagkaroon ka ba ng dilated eye exam sa loob ng nakaraan na 12 buwan? Ito ay kapag pinapatakan ang iyong mata ng eye drops at maaring nagdulot ng pansmantalang paglabo ng paningin o pagiging sensitibo sa ilaw
   1. Oo
   2. Hindi
7. *(if b for 5)* Sa iyong buong buhay, nagkaroon ka ba ng dilated eye exam? Ito ay kapag pinapatakan ang iyong mata ng eye drops at maaring nagdulot ng pansmantalang paglabo ng paningin o pagiging sensitibo sa ilaw
   1. Yes
   2. No
8. Piliin ang lahat ng naaangkop. Alin sa mga sumusunod ay ang iyong health insurance?
   1. No insurance
   2. Medicare
   3. Medicaid (Medi-Cal)
   4. Ibang insurance na galing sa gobyerno (VA, Tricare, etc)
   5. Ang aking insurance ay galing sa aking employer
   6. Ang aking insurance ay pribadong binibili
   7. Ang aking insurance ay binili mula sa exchange
   8. Hindi alam/ Hindi sigurado
9. Meron ka bang health insurance coverage para sa pag-papatingin ng mata o para sa pagbili ng salamin?
   1. Oo, meron
   2. Wala
   3. Hindi alam/ Hindi sigurado
10. Piliin mula sa mga sumusunod kung paano nakaapekto ang inyong health insurance sa kadalasan ng inyong pagpacheck-up ng mata. 'Dahil sa sitwasyon ng aking health insurance...'
    1. Mas bihira akong nakakpagpacheck up kumpara sa aking gugustihin
    2. Hindi pa ako nakakapagpacheck-up ng mata dahil ang pagpapatingin ng mata ay hindi kasama sa aking health insurance
    3. Nakakapagpacheck-up ako ng mata kapag aking gugustuhin.
    4. Hindi nakaka-apekto ang aking health insurance sa kadalsan ng aking check-up sa mata dahil binabayad ko ito ng buo (pay out-of-pocket).
11. Piliin ang lahat ng naaangkop. Nasabihan ka na ba ng iyong doktor na ikaw ay nagkaroon na ng…
    1. Diyabetis?
    2. Mataas na prisyon/hypertension?
    3. Stroke?
    4. Diyabetis na nakakaapekto ng ugat sa mata o na meron kang diabetic retinopathy or diabetic eye disease
    5. Macular degeneration (AMD) o senile macular degeneration ng retina -- ito ang pagkasira ng macular sa likod ng mata
    6. Katarata sa alinman sa mata
    7. Glaucoma sa alinman sa mata
    8. Ptosis o droopy eyelid, nakababa o hindi pantay ang isang talukap ng mata
12. *(if a for 10)* Kasalukuyan ka bang umiinom ng gamot para sa mataas na blood sugar?
    1. Oo
    2. Hindi
13. *(if a for 10)* Gumagamit ka ba ng insulin para sa blood sugar?
    1. Oo
    2. Hindi
14. *(If b for 10)* Kasalukuyan ka bang umiinom ng gamot para sa mataas na prisyon?
    1. Oo
    2. Hindi
15. Gaano ka kadalas naninigarilyo ng tabako?
    1. Araw-araw
    2. paminsan-minsan
    3. Hindi ako sumisindi ng tabako
16. *(if b for 14)* Dati ka bang naninigarilyo ng tabako araw-araw?
    1. Oo
    2. Hindi
17. *(if c for 14)* Dati ka bang naninigarilyo ng tabako?
    1. Oo, araw-araw
    2. Oo, paminsan-minsan
    3. Hindi ako sumisindi ng tabako, kahit dati.

**Demographic questions**

1. Kasarian
   1. Lalaki
   2. Babae
   3. Iba______________
2. Ano ang kasalukuyang estado ng iyong trabaho?
   1. Full-time na nagtatrabaho. Ang trabaho ko ay: ______________
   2. Part-time na nagtatrabaho. Ang trabaho ko ay: ______________
   3. Hindi nagtatrabaho
   4. Self-employed
   5. Disabled
   6. Estudyante
   7. Sa militar
   8. Nagretiro
3. Saan ka pinanganak?
   1. Sa Pilipinas
   2. Sa Amerika
   3. Hindi sa Amerika at hindi sa Pilipinas
4. Edukasyon
   1. Walang nakumpleto na pag-aaral
   2. Iilang taon ng high school pero walang diploma
   3. High school diploma
   4. Trade/technical/vocational training
   5. Associate's degree
   6. Iilang taon sa colegio, ngunit mas mababa sa isang 4-taong degree
   7. Bachelor's degree or kapatay nito ang nakumpleto
   8. Nagumpisa o nakumpleto ang graduate school
5. Kabuuang kita kada taon
   1. Mas mababa sa $25,000
   2. $25,000 to $49,999
   3. $50,000 to $74,999
   4. $75,000 to $99,999
   5. $100,000 to $124,999
   6. $125,000 to $149,999
   7. $150,000 to $174,999
   8. $175,000 to $199,999
   9. $200,000 at pataas
6. Saan namin pwedeng ipadala ang inyong gift card? (paki sulat ang inyong email address)
7. Piliin ang lahat ng mga naangkop. Pwede ba namin kayong sulatan sa darating na mga araw para sa mga karagdagang tanong sa aming pagsusuri?
   1. Oo (email)
   2. Oo (phone number)
   3. Hindi, hindi ko na nais tumulong sa karagdagang pagsusuri.

Para sa mga sumusunod na pangugusap, mangyaring piliin ang sagot na naaangkop na sagot para sa iyo.
Kung mobile phone ang gamit niyo para sa survey na ito, gawin ang sumusunod na mga tanong nang naka-pahalang ang iyong telepono para makita ang mga pagpipilian na sagot)

**Options for the following statements were the following:* Mga wikang Pilipino lamang, Karamihan ay wikang Pilipino kumpara sa Ingles, Parehong pantay na wikang Pilipino at Ingles, Karamihan ay Ingles kumpara sa wikang Pilipino, Ingles lamang

1. Mga (mga) wika na ginagamit sa bahay
2. Wika ng aking pagbabasa at pagsasalita
3. Wika na aking ginagamit kausap ang aking mga kaibigan
4. Wika ng mas gusto kong pinapanood na mga pelikula, TV, at media
5. Wika na aking ginagamit sa pag-iisip
6. Wika na aking gamit sa pasgsasalita noong ako ay bata pa
7. Wika ng mga programa sa TV na aking karaniwang pinapanood

**Options for the following statements were the following: Lahat ay mga Pilipino, Karamihan ay Pilipino kumpara sa mga Amerikano, Halos kalahati ay Pilipino at kalahit ay Amerikano, Karamihan ay Amerikano kumpara sa mga Pilipino, Lahat ay mga Amerikano*

1. Ang etniko (ethnicity) ng mga bisita o mga taong iyong binibisita
2. Ang etniko (ethnicity) ng mga matalik mong kaibigan
3. Ang etniko (ethnicity) ng mga tao sa karamihan ng iyong pinupuntahan na pagtitipon
4. Etniko (ethnicity) ng mga kaibigan ng iyong mga anak (kung hindi naaangkop, mangyaring iwanan ng blangko)
